# From Bench to Brain: Translating EV and Nanocarrier Research into Parkinson’s Disease Therapies

**DOI:** 10.3390/biology14101349

**Published:** 2025-10-02

**Authors:** Barathan Muttiah, Nur Atiqah Haizum Abdullah

**Affiliations:** 1 Department of Medical Microbiology and Immunology, Faculty of Medicine, Universiti Kebangsaan Malaysia, Cheras, Kuala Lumpur 56000, Malaysia; 2 Department of Tissue Engineering and Regenerative Medicine, Faculty of Medicine, Universiti Kebangsaan Malaysia, Cheras, Kuala Lumpur 56000, Malaysia

**Keywords:** Parkinson’s disease, extracellular vesicles, α-synuclein, mesenchymal stem cells, drug delivery, nanocarriers

## Abstract

Parkinson’s disease (PD) is a disabling neurodegenerative disorder that is defined by progressive loss of dopaminergic neurons in the substantia nigra, deposition of α-synuclein aggregates, and chronic neuroinflammation. While symptomatic therapies have evolved, disease-modifying therapies remain elusive. Extracellular vesicles (EVs), particularly those derived from mesenchymal stem cells (MSC-EVs), have emerged as promising therapeutic agents because they possess a natural ability to cross the blood–brain barrier and deliver bioactive cargo. Herein, we review the dual-edged function of EVs in PD pathogenesis: facilitating the transfer of toxic α-synuclein while also conferring neuroprotective signals through MSC-EVs. We outline the mechanisms of MSC-EV-mediated neuroprotection that include the regulation of oxidative stress, neuroinflammation, and autophagy. We also emphasize new nanocarrier systems designed to bypass delivery challenges in PD therapy. While preclinical studies are extremely encouraging, significant issues regarding scalability, standardization, and clinical translation must be resolved before realizing the ultimate therapeutic potential of EV-based and nanocarrier-based approaches to PD.

## Simple Summary

PD is marked by dopamine-secreting neuron loss, α-synuclein deposition, and chronic brain inflammation, with no disease-stopping or disease-slowing therapies on the horizon. EVs, especially those derived from mesenchymal stem cells, hold promise since they can cross the blood–brain barrier and deliver brain cell-protective molecules. Interestingly, EVs have a dual role in PD: they can spread toxic α-synuclein between cells, but they also can provide neuroprotection by alleviating oxidative stress, modulating inflammation, and enhancing cell survival through autophagy. In parallel, nanocarrier platforms like liposomes and nanoparticles are being designed to improve drug delivery to the brain. Both EVs and nanocarriers, however, are limited by large-scale production, reproducible quality, and translational medicine to the clinic. Combined, these approaches have promising potential for potential PD therapies.

## 1. Parkinson’s Disease (PD)

Parkinson’s disease (PD) is a chronic, progressive neurodegenerative disorder with initial involvement of the motor system, presenting as bradykinesia, resting tremor, rigidity, and postural instability [1]. It is pathologically characterized by progressive loss of dopaminergic neurons in the substantia nigra pars compacta, resulting in reduced dopamine in the striatum and hence compromising the basal ganglia’s function to regulate voluntary movement [2]. This leads to motor symptoms, such as shuffling gait, micrographia, and masked face. In addition to these motor impairments, PD also includes a wide range of non-motor symptoms such as depression, anxiety, sleep disturbance, anosmia, constipation, cognitive impairment, and autonomic dysfunction, all of which severely impair quality of life [3]. The presence of Lewy bodies, an abnormal intracellular inclusion of α-synuclein protein that serves as the disease’s signature pathology [4]. Despite the advancements in symptom treatment with pharmacologic medications such as levodopa, dopamine agonists, monoamine oxidase B (MAO-B) inhibitors, and surgery such as deep brain stimulation, there is no cure available, and treatment is directed primarily at improving function and preventing symptom progression [5].

Based on the most recent available statistics, more than 10 million individuals coexist with PD globally, with more than 8.5 million reported by the World Health Organization for the year 2019, a number that has nearly doubled over the past 25 years [6]. The Global Burden of Disease analysis for the year 2021 has estimated approximately 11.77 million cases globally, a 274% rise from 1990. The age-standardized prevalence rate, too, rose by 28%, while disability-adjusted life years (DALYs) for PD rose by 81% since the year 2000, and mortality rates doubled more than two times, reflecting the mounting social and healthcare burden [7]. Aging remains the most potent risk factor, and prevalence is approximately 1% in individuals aged over 60 years. With increased life expectancy globally, the at-risk population keeps rising, fueling the rising trend in PD incidence [8]. Geographic distribution of the disease is quite disparate, and the highest prevalence is seen in Latin America and the Caribbean (1081 per 100,000), East Asia and the Pacific (688 per 100,000), and Europe and Central Asia (464 per 100,000) and is significantly low in Sub-Saharan Africa (49 per 100,000) and South Asia [9]. PD prevalence in the future will grow by 76% globally in the year 2050, and the burden will be borne mostly by the East Asian and South Asian nations [8,9]. The national prevalence will be the highest in Spain, China, and Andorra. The younger age groups in some African nations could have reduced absolute burdens but elevated percentage increases [9]. In the United States, new PD diagnoses have increased annually from 60,000 to some 90,000, a 50% growth, highlighting national healthcare importance [9,10].

## 2. PD Pathogenesis

PD pathogenesis is choreographed by a multistep concert of molecular and cellular mechanisms, based on α-synuclein misfolding. α-synuclein is a presynaptic protein that controls synaptic vesicles and can misfold to β-sheet–rich fibrils that accumulate to form Lewy bodies and Lewy neurites, a PD’s pathological hallmarks [11]. This misfolding is further potentiated by mutations such as A30P, A53T, and E46K, as well as by post-translational modifications such as phosphorylation at Ser129, nitration, and truncation [12]. Once they aggregate, α-synuclein impairs the docking and release of synaptic vesicles and the dopamine neurotransmission. These aggregates can also propagate in a prion-like fashion, being sequestered into extracellular vesicles (EVs), secreted beyond the cell, and engulfed by nearby neurons or glial cells, where they seed further aggregation and spread pathology across connected brain networks [13,14]. Meanwhile, dysregulation of the metal ion is the core of PD pathogenesis and progression in that it has its effect through a multi-component interrelated system involving oxidative stress, protein misfolding and aggregation, cell death of the neurons, and neuroinflammation [15]. Overload of iron is one of the best-characterized and most crucial features of metal ion derangement in PD. Excess iron preferentially accumulates in susceptible areas of the brain, particularly the substantia nigra pars compacta, where dopaminergic neuronal loss occurs gradually in PD [15,16]. Iron overload is associated with both the severity of motor dysfunction as well as cognitive impairment. Molecularly, iron catalyzes the Fenton reaction to produce highly reactive hydroxyl radicals from hydrogen peroxide and thereby to reactive oxygen species (ROS) [16]. The elevated ROS induce lipid peroxidation, DNA damage, and protein oxidation, overloading neuronal antioxidant defenses and triggering oxidative stress. This cascade promotes enhanced mitochondrial dysfunction and triggers ferroptosis, an iron-catalyzed process of cell death that involves the accumulation of lipid peroxides [17]. Enzymes like acyl-CoA synthetase long-chain family member 4 (ACSL4) propel the formation of polyunsaturated fatty acid-enriched phospholipids, susceptible to peroxidation, sensitizing neurons further for ferroptosis [18]. Ironically, ferritin, the principal iron-storage protein sequestering free iron in non-toxic form, is often decreased in PD brains despite iron overload. This decrease undermines iron buffering capacity, allowing free iron to participate in redox reactions more freely and thereby increasing oxidative damage. Such imbalance between iron accumulation and ferritin decrease not only accelerates neurodegeneration but also may serve as a potential useful biomarker for measuring disease progression [19]. Iron deposition, in particular, is a common finding in the substantia nigra of PD patients and is closely associated with oxidative damage and neuronal vulnerability [20]. Increased intracellular calcium also contributes to aggregation by inducing the assembly of RNA G-quadruplex scaffolds that permit α-synuclein to aggregate. The resulting oxidative and ionic derangement is a potent inducer of neuronal damage and death [19,20]. With the exception of iron, deregulation of several other metals, such as copper and manganese, participates in PD. Elevated exposure to manganese, common in certain workplaces, is known to cause manganism, a Parkinsonian syndrome [21]. Manganese interferes with dopaminergic neuronal function through interference with calcium and potassium ion channels, blockade of mitochondrial function, and increases in oxidative stress. Similarly, copper, required for several enzymatic processes, when uncontrolled, is involved in ROS generation and promotes worsening of α-synuclein aggregation [22]. Lead, though less studied, is suspected to be implicated in neurodegeneration via oxidative and inflammatory pathways. Metal ion imbalance also leads to neuroinflammation, a characteristic of PD. Free metal ions activates microglial cells, the innate immune cells of the brain, to release pro-inflammatory cytokines and nitric oxide that expand neuronal damage [23]. Toxic interactions among metals and α-synuclein induce cellular senescence, a state of irreversible growth inhibition with pro-inflammatory secretions that damages nearby neurons. This chronic inflammatory milieu spawns a feed-forward mechanism of neurodegeneration [24].

In addition, autophagy–lysosome dysfunction is an important pathogenic process in PD, and mutations in leucine-rich repeat kinase 2 (LRRK2), GBA, and ATPase Type 13A2 (ATP13A2) illustrate how genetic dysregulation of lysosomal biology facilitates neurodegeneration [25,26]. LRRK2 plays a particularly critical role in familial and idiopathic PD. Mutations in LRRK2, and most notably the prevalent G2019S mutation, are the most frequent cause of autosomal dominant PD and occur in a subgroup of sporadic cases [27]. LRRK2 codes for a large multidomain protein with kinase and GTPase activity, and disease-causing mutations are likely to increase kinase activity, leading to hyperphosphorylation of downstream targets such as Rab GTPases. This dysregulation perturbs vesicle trafficking, dismantles autophagy and mitophagy, destabilizes cytoskeletal dynamics, and contributes to mitochondrial dysfunction [28]. In addition, LRRK2 is highly expressed in immune cells like microglia, where mutant isoforms amplify inflammatory signaling and accelerate neurodegeneration. Pathological LRRK2 is also microtubule interacting, with interruption of their dynamics and intracellular transport [29]. These pathological activities cumulatively promote α-synuclein aggregation, oxidative stress, and dopaminergic neuronal degeneration. Because of its enzymatic activity and multiplicity of function, LRRK2 is considered a primary therapeutic target, and several methodologies are underway at the moment that target the kinase activity [28,29]. The GBA gene encodes the lysosomal enzyme glucocerebrosidase (GCase), responsible for cleaving glycosphingolipids such as glucosylceramide. GBA mutations are the most common genetic risk factor for PD, occurring in roughly 5–15% of patients [30,31]. Reduced GCase activity results in lysosomal impairment and impaired autophagic-lysosomal degradation, resulting in α-synuclein accumulation and the development of Lewy bodies, a definitive sign of PD pathology [32]. In addition, GCase deficiency breaks lipid homeostasis, resulting in glucosylceramide and glucosylsphingosine accumulation, which also increase α-synuclein aggregation and neurotoxicity. Mutant GCase can also become sequestered in the endoplasmic reticulum (ER), activating ER stress and unfolded protein response (UPR), while other processes such as mitochondrial dysfunction and neuroinflammation add to neuronal injury. Both loss-of-function (deficient GCase activity) and toxic gain-of-function (toxicity of abnormally folded protein) mechanisms contribute to GBA-PD [33,34]. On a clinical level, GBA-PD is typically marked by younger age at onset, faster progression, and greater cognitive impairment compared to sporadic PD. In parallel, these mechanisms overlap to mediate dopaminergic neuron degeneration in the substantia nigra and the pathogenesis of PD motor and non-motor symptoms [35]. ATP13A2, also known as PARK9, is a P5-type lysosomal ATPase with mutations that have been linked to Kufor-Rakeb syndrome, an autosomal recessive, juvenile-onset form of PD characterized by parkinsonism [36]. ATP13A2 plays a pivotal role in the function of lysosomes as it is a lysosomal H^+^/K^+^ pump essential for acidification, maturation of proteolytic enzymes, autophagy, and degradation of α-synuclein, the major PD pathologic protein [37]. ATP13A2 deficiency or failure perturbs lysosomal clearing mechanisms, leading to lysosomal dysfunction, α-synuclein aggregation, imbalance of metal ion homeostasis (specifically, zinc), mitochondrial dysfunction, and impaired autophagy [38]. These deficits each contribute to dopaminergic neuronal loss in the substantia nigra pars compacta and therefore induce motor and non-motor symptoms of PD. In addition, ATP13A2 deficiency further increases neuroinflammation by activating microglia and astrocytes and inducing neurodegeneration [39]. With its central position in neuronal and lysosomal homeostasis, restoration of ATP13A2 function or modulation of ATP13A2-related pathways is a therapeutic potential to reduce α-synuclein burden, mitigate neuroinflammation, and sustain dopaminergic neuron survival in PD. Overall, these genes have distinct functions, but their pathogenic alleles converge on one pathway: compromised lysosomal acidification, defective enzymatic degradation, and impaired removal of misfolded proteins such as α-synuclein. This disruption of proteostasis triggers toxic protein accumulation, mitochondrial stress, and progressive dopaminergic neuronal death [40,41,42].

Proteasomal function is also compromised, in part by inhibition from deteriorated protein and oxidative damage, and reduced turnover of compromised cellular structures. The ubiquitin–proteasome system (UPS) is responsible for selectively degrading short-lived, damaged, or misfolded proteins that are tagged with polyubiquitin chains [43]. In PD, oxidative stress, α-synuclein aggregation, and environmental toxins converge to disrupt this pathway, resulting in the progressive accumulation of toxic proteins and organellar damage [44]. Oligomeric α-synuclein assumes conformations that bind to proteasomal subunits allosterically, closing the axial gates of the 20S core. This prevents the 19S regulatory complex from unfolding and threading ubiquitinated proteins into the proteolytic chamber for degradation [45]. A mechanical block to the proteasome reduces the turnover of damaged proteins, increasing proteotoxic stress and contributing to the formation of Lewy bodies. Because these inclusions can capture parts of the proteasome as well, this is a functional and structural inhibition [46]. At the same time, reduced levels or impaired activity of molecular chaperones such as heat shock proteins Hsp70 and Hsp90 are increasingly implicated in the pathogenesis of PD. These chaperones are key components of the protein quality control (PQC) system that maintains proteostasis by promoting correct folding of newly synthesized proteins, refolding of misfolded proteins, and directing irreversibly misfolded proteins to degradation pathways [47]. In PD, both chaperones can bind α-synuclein, reducing its tendency to aggregate. Hsp70 was shown to prevent α-synuclein fibril formation and toxicity, and Hsp90 may indirectly regulate α-synuclein turnover via its client proteins [48,49]. Neuroinflammation becomes both a consequence and a cause of PD pathology. Aggregated α-synuclein activates microglia via Toll-like receptors (TLR2 and TLR4), inducing the production of pro-inflammatory cytokines such as TNF-α, IL-1β, and IL-6, which further damage neurons [50]. Reactive astrocytes abolish their neuroprotective roles, instead contributing to excitotoxicity and oxidative stress [51]. Peripheral immune cells, particularly monocytes, may enter the brain and amplify inflammation, and some data suggest that modified α-synuclein may act as a neoantigen, triggering autoimmune destruction of dopaminergic neurons [52].

Mitochondrial dysfunction in PD results from various interconnected mechanisms that lead to the death of dopaminergic neurons in the substantia nigra. The central defect is complex I deficiency in the electron transport chain, which lowers ATP production and elevates the ROS, causing oxidative stress and damage to mitochondrial DNA, proteins, and lipids [53]. A recent study on a genetic approach has shown that disruption of complex I function specifically in dopaminergic neurons is sufficient to produce a progressive Parkinsonism [54]. Mitochondrial dysregulation of dynamics with overactive fission and impaired fusion creates fragmented and dysfunctional mitochondria, while faulty mitophagy due to PINK1 and Parkin mutations allows defective mitochondria to accumulate. Abnormal calcium homeostasis further adds to neuronal stress by promoting calcium overload, and chronic oxidative stress causes mitochondrial DNA mutations leading to compromised biogenesis [55]. On the other hand, pathogenic α-synuclein aggregates directly obstruct complex I function and cause mitochondrial fragmentation. Together, these mechanisms cause bioenergetic failure, oxidative stress, and the activation of cell death mechanisms that initiate the selective degeneration of dopaminergic neurons in PD [56].

**Figure 1:**
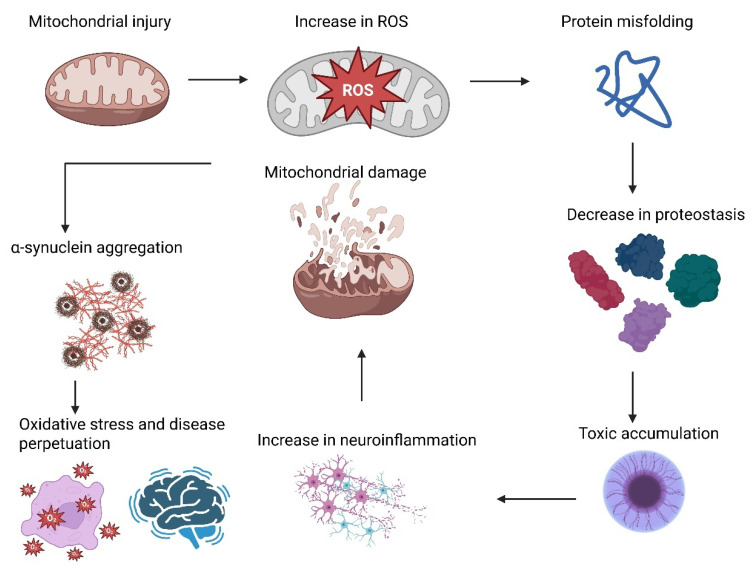
PD vicious cycle pathways. (1) Primary mitochondrial damage increases ROS generation, inducing α-synuclein misfolding. (2) Aggregated α-synuclein disrupts proteasomal/autophagic clearance (proteostasis failure). (3) Resulting oxidative stress and protein aggregates activate microglia, sustaining chronic neuroinflammation.

Gut microbiota dysbiosis alters metabolite profiles including short-chain fatty acids (SCFAs) that can modulate neuroinflammation and α-synuclein aggregation [57]. Patients with PD have reduced microbial diversity, a loss of SCFA–producing microbes, and an overabundance of pro-inflammatory taxa, deviations that are evident even in prodromal stages with gastrointestinal symptoms. In addition, changes in microbial metabolites to pro-inflammatory metabolites and different availability of neurotransmitter precursors exacerbate PD pathology and symptoms [57]. Synthetic compounds, including MPTP, environmental toxins, and specifically pesticides such as paraquat and rotenone, also disrupt mitochondrial complex I function and promote α-synuclein misfolding [58]. Lastly, these routes converge into a vicious circle, mitochondrial injury promotes ROS generation, which increases protein misfolding; impaired proteostasis facilitates toxic species accumulation; neuroinflammation exacerbates mitochondrial injury; and α-synuclein aggregation gives rise to increased inflammation and oxidative stress [59]. This positive feedback ensures disease perpetuation, hence the need for therapeutic options that target multiple pathologic mechanisms instead of one mechanism. Determination of these multifaceted etiologic factors is important for effective deployment of prevention measures, diagnostic tools, and disease-modifying therapies in the future. Figure 1 shows the pathological cascade in PD.

## 3. The Involvement of EV in PD Pathogenesis

EVs have emerged as central protagonists in the pathogenesis of PD, acting both as vectors of pathological cargo and amplifiers of neurodegenerative pathways [60]. These nanosized, membrane-bound particles secreted by neurons, glia, and other cells, once considered passive waste products of cellular activity, are now considered to play an active role in the propagation of disease [61]. The hallmark of PD is the misfolding and aggregation of α-synuclein which builds up in Lewy bodies and Lewy neurites [62]. Specifically, monomeric α-synuclein usually present in normal state, regulates synaptic vesicle release; released in exosomes, activate microglia, and enter postsynaptic cells [63]. Meanwhile, oligomeric α-synuclein is present in small aggregates, highly toxic; it is synthesised with dopamine/DOPAL, destabilises membranes, damages mitochondria, destroys lipid rafts, and spreads through EVs [64]. Lastly, fibrillar α-synuclein appears in large aggregates (Lewy bodies); aggregate vesicles, lyse membranes, activate microglia, and seed further aggregation, spreading PD pathology [65].

EVs are involved in the prion-like spread of this pathogenic protein through a selective cargo packaging mechanism, extracellular secretion, target cell-specific uptake, and intracellular seeding [66]. Aggregated α-synuclein oligomers and fibrils are linked to EV lipid membranes, promoting their incorporation into exosomes and microvesicles, often in multivesicular bodies (MVBs) before exocytosis [67]. Mutations in the SNCA gene, such as A53T, enhance α-syn aggregation and its association with EVs, and lysosomal dysfunction which is universal in PD further increases the secretion of α-synuclein–laden EVs, as degradation pathways are impaired [68]. α-synuclein-containing EVs released migrate through the extracellular space and, although they constitute a minority of secreted α-synuclein, are disproportionately effective at cell-to-cell transmission of pathology [69]. These EVs are internalized by recipient glia and neurons primarily via clathrin-mediated endocytosis, macropinocytosis, or membrane fusion with high-affinity binding facilitated by receptors such as lymphocyte activation gene 3 (LAG3) and heparan sulfate proteoglycans (HSPGs), which are biased towards fibrillar and phosphorylated α-synuclein species [70]. Upon internalization, EVs fuse with late endosomes or lysosomes, releasing their α-synuclein cargo into the cytosol, where it seeds the misfolding and aggregation of resident α-synuclein, thereby amplifying neurodegenerative changes [71]. Notably, EV-mediated α-synuclein spread is not limited to the central nervous system; EVs from peripheral sources, such as erythrocytes, can carry α-synuclein across the blood–brain barrier under inflammatory conditions, inducing microglial activation and promoting PD pathology further [72].

In addition to protein spread, EVs also play a key role in the perpetuation of neuroinflammation, a second key driver of PD progression. Activated microglia and astrocytes secrete EVs enriched with pro-inflammatory cytokines, ROS–producing enzymes, and damage-associated molecular patterns (DAMPs), which cause a feed-forward loop of glial activation and oxidative stress [73]. Genetic mechanisms also converge on these processes, with mutations in LRRK2, VPS35, and GBA1 disrupting endosomal–lysosomal homeostasis and increasing the release of EVs that contain pathogenic protein and inflammatory mediators [74,75,76]. EVs in PD also have a heterogeneous molecular payload other than misfolded α-synuclein, which includes regulatory RNAs, proteins, and enzymes that cumulatively impact disease progression. Among small non-coding RNAs, microRNAs (miRNAs) are important regulators of gene expression, and several have been directly involved in PD pathogenesis [77,78]. For example, miR-7 and miR-153 are post-transcriptional regulators of SNCA, the α-synuclein-encoding gene, thereby controlling α-synuclein synthesis and accumulation [79]. Under pathological conditions, EVs may carry varying amounts of these miRNAs, inducing dysregulation of α-synuclein in acceptor neurons and favoring aggregation. In addition, pro-inflammatory miRNAs such as miR-21 and miR-155, which are frequently enriched in EVs of activated glia, can modulate microglial activation and cytokine release, exacerbating neuroinflammation and neuronal stress [80,81]. EV lncRNAs also have important functions in PD pathology, often by modulating autophagy and protein clearance pathways [82]. Certain dysregulated lncRNAs impair the autophagic–lysosomal pathway, reducing the degradation capacity for aggregated α-synuclein and favoring protein accumulation [83]. For instance, pathogenic EV-associated lncRNAs can downregulate autophagy-related genes or alter mTOR signaling to tip the balance towards impaired proteostasis. This imbalance not only augments α-synuclein accumulation but also aggravates downstream toxic pathways such as mitochondrial dysfunction and oxidative stress [84]. EV protein cargo is no less significant. Aside from carrying α-synuclein fibrils themselves thus acting as seeds for propagating pathological aggregation, EVs often carry enzymes and signal-transducing proteins that modulate mitochondrial dynamics, apoptosis, and inflammatory cascades [85]. While some EVs are enriched in mitochondrial proteins reflecting organelle damage or stress, others carry pro-inflammatory mediators that can activate glia or precondition neurons for oxidative insults [86]. In addition, EV membrane components, such as gangliosides, may enhance the nucleation potential of α-synuclein aggregates, rendering them more pathogenic upon transmission to acceptor cells [87]. Clarifying the precise roles of each cargo type will be essential for the development of EV-based diagnostics and targeted therapies capable of intercepting PD progression at multiple molecular levels.

Beyond α-syn aggregation, several other biomarkers have been found that may aid in the diagnosis and follow-up of PD. The most studied protein markers are alterations in autophagy-related proteins like LC3B, Beclin1, and LAMP-2 in cerebrospinal fluid (CSF) as indicators of early dysfunctions in the autophagy–lysosomal pathway [88]. Similarly, amyloid-β 1–42 (Aβ1–42), tau, and phosphorylated tau proteins, which are commonly associated with Alzheimer’s disease, have been found in PD with reduced Aβ42 levels correlating with the progression of non-motor symptoms, including freezing of gait [89]. Certain cytoskeletal and inflammatory markers such as neurofilament light chain (NfL) and glial fibrillary acidic protein (GFAP) in CSF and serum indicate axonal damage and astroglial activation, respectively [90]. Additionally, post-translationally modified α-synuclein in red blood cells including phosphorylated, nitrated, and glycated types correlate with disease severity and provides a minimally invasive biomarker candidate [91]. Genetic and molecular events also make it easy to discover PD biomarkers; for example, overexpressed chemokines such as CCL28 are an indication of aberrant immune activation, while truncated and phosphorylated α-synuclein fragments have distinctive pathological signatures to enhance diagnostic specificity [92]. Furthermore, PD-associated gene mutations in LRRK2 and GBA are linked to altered enzymatic activities being intensely researched as both biomarkers and therapeutic targets [93]. Neuroimaging biomarkers also amplify diagnostic potential: dopaminergic PET and SPECT with DaTscan can quantify nigrostriatal loss, whereas novel tracers to α-syn aggregates, mitochondrial damage, or microglial activation are under development [94]. Structural and functional MRI techniques, including diffusion tensor imaging and resting-state fMRI, also reveal network-level and microstructural changes tied to both motor and cognitive impairment [95].

In addition to central markers, peripheral biofluids and tissues have provided us with easily accessible windows for diagnosis. Altered cytokine profiles, oxidative metabolites of stress, and changes in plasma and serum proteomes reflect systemic pathology, while phosphorylated α-syn aggregates have been found in submandibular gland and skin biopsies that offer minimally invasive diagnostic tools [96]. Collectively, these heterogeneous biomarkers complement α-syn aggregation with molecular, immunologic, imaging, and peripheral signatures that enhance early diagnosis, enable differential diagnosis, and allow for more precise monitoring of disease course and therapeutic response.

**Figure 2:**
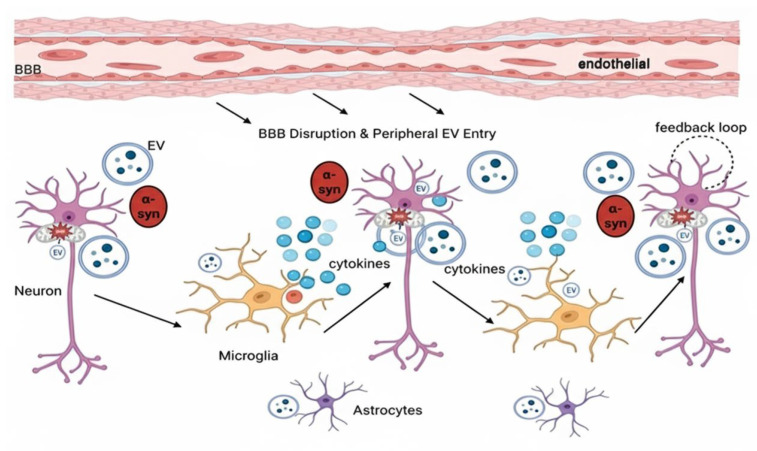
The action of EVs contribute to PD pathologies.

Figure 2 EVs contribute to PD pathology. Parkinson’s disease pathology can be imagined as a cascade of several events triggered by environmental or genetic insults like pesticide exposure, heavy metals, gut microbiome change, or SNCA, LRRK2, GBA, or VPS35 mutations that impair mitochondrial function, increase oxidative stress, and promote α-synuclein misfolding. EV release carrying toxic α-syn early permits prion-like transmission to neighbouring neurons and glia, seeding further aggregation. Microglial uptake of α-syn–laden EVs induces inflammatory signaling (NF-κB, NLRP3) and cytokine release, augmenting neuronal damage. Activated inflammation disrupts the blood–brain barrier, facilitating bidirectional trafficking of peripheral and central EVs, further increasing α-syn load. It promotes a feedback loop of protein accumulation, oxidative damage, and neuroinflammation, leading to Lewy body development and dopaminergic neuron degeneration. By the time motor signs of tremor, rigidity, and bradykinesia appear that typically with accompanying cognitive impairment, about 60% of nigral dopamine neurons are lost, but EV-mediated pathology still underlies neurodegeneration.

## 4. Therapeutic Mechanisms of MSC-EVs in PD

Mesenchymal stem cell–derived extracellular vesicles (MSC-EVs) are therapeutic in PD by virtue of a concerted set of biological mechanisms that strain both the symptomatic and underlying pathologic features of the disorder [97]. One of the most significant properties is the ability to cross the blood–brain barrier (BBB) due to their nanoscale size (typically 40–100 nm) and their specific surface molecules like tetraspanins, integrins, and chemokine receptors like CCR2 and CXCR4, MSC-EVs can efficiently cross the BBB. This opens the prospect for direct delivery of therapeutic cargo to impacted brain regions, including the substantia nigra and striatum, where dopaminergic neurons progressively degenerate in PD [98,99].

Once in the brain, MSC-EVs act in a neuroprotective manner by delivering a range of bioactive molecules such as proteins, enzymes, growth factors, and microRNAs that support neuronal function and survival [100]. Their cargo is heterogeneous and functionally complementary in nature such that they are able to address multiple pathological processes in PD simultaneously. The most significant cargo content of MSC-EVs is their arsenal of proteins and enzymes with inherent neuroprotective and restorative functions. Notably, they carry neurotrophic factors such as brain-derived neurotrophic factor (BDNF) and glial cell line-derived neurotrophic factor (GDNF), which are crucial for dopaminergic neuron survival, neurite outgrowth, and synaptic homeostasis [101]. Additionally, there are enzymes like catalase and superoxide dismutase (SOD), which inhibit ROS and abate oxidative stress, a leading force of dopaminergic neuron degeneration in PD [102]. There are also signaling molecules among the proteins that activate pro-survival pathways, such as PI3K/Akt and MAPK/ERK, that enhance a cellular environment conducive to repair and regeneration [103]. These proteins are complemented by growth factors that enhance neuronal growth, differentiation, and synaptic plasticity. Through the enhancement of dendritic spine formation, branching of axons, and synapse stability, neurons are engaged in the repair of disrupted neural networks in the basal ganglia and motor cortical circuits. These network-level adjustments are necessary for the recovery of motor coordination, balance, and cognitive function that are progressively impaired as PD advances [104,105]. Perhaps the most promising cargo of MSC-EVs are miRNAs, a short, non-coding RNAs that regulate gene expression post-transcriptionally in target cells. For example, miR-627-5p suppresses FTO demethylase and α-synuclein expression by m6A RNA methylation, and miR-181a-2-3p mitigates oxidative stress through EGR1 targeting and NOX4/p38 MAPK pathway regulation [106]. Similarly, miR-124 diminishes neuroinflammation through the regulation of autophagy- and inflammation-related factors p62 and p38 MAPK [107]. Other miRNAs, including miR-204-5p and miR-497-5p, modulate autophagy and apoptosis with context-dependent regulation of neuronal survival [108]. Together, these miRNAs target complementary PD-pathway mechanisms—enhancing autophagic clearance of aggregated α-synuclein, suppressing apoptosis, and preventing neuroinflammation—to preserve dopaminergic neuron integrity and slow disease progression [109]. At the same time, MSC-EVs also exert potent immunomodulatory activities, suppressing neuroinflammation through downregulating the expression of key pro-inflammatory cytokines such as tumor necrosis factor-alpha (TNF-α), interleukin-6 (IL-6), and interleukin-1β (IL-1β). Through modulating microglial activation and peripheral immune infiltration, MSC-EVs limit inflammatory damage that promotes neurodegeneration [110].

Another essential therapeutic function of MSC-EVs in PD is their effect on α-synuclein pathology. There is increasing evidence that EV cargo can promote the degradation or elimination of pathological α-synuclein aggregates through autophagy induction and proteostasis maintenance [111]. This activity has the potential to disrupt the propagation of toxic α-synuclein species across inter-connected neuronal networks, a key driver of disease progression [112]. MSC-EVs also carry regulatory RNAs with epigenetic control such as microRNAs and siRNAs that can potentially influence gene expression relevant to disease. For example, inhibition of the N6-methyladenosine fat mass and obesity-associated protein through EV-mediated miRNAs have been shown to reduce α-synuclein expression and protect dopaminergic neurons from apoptosis [113]. Along with protein aggregation, MSC-EVs act on mitochondrial dysfunction and oxidative stress, two hallmark features of PD pathophysiology. They contain antioxidant enzymes and mitochondria-stabilizing factors that enhance cellular energy metabolism, reduce ROS, and restore homeostatic signaling pathways relevant to neuronal resilience [114]. These cellular effects are mirrored in functional improvement: PD animal models treated with MSC-EVs show measurable improvement in motor coordination, limb agility, and balance, with effects frequently sustained for weeks after treatment cessation [115]. MSC-EVs have been deemed a new, cell-free therapeutic strategy for PD, taking advantage of their ability to cross the blood–brain barrier and deliver neuroprotective factors. Preclinical data consistently show efficacy in animal models, while human clinical translation is still in its early stages, with no reported results from currently enrolled or completed clinical trials in PD as of mid-2024 with key challenges including standardization, regulatory approval, and long-term safety evaluation [115,116]. Table 1 shows that MSC-EVs offer multi-modal therapy (neuroprotection + immunomodulation + α-synuclein clearance).

## 5. EVs from Non-MSC in PD Therapy

While MSC-EVs have garnered most of the attention in PD investigations, other sources of EVs are also under investigation, each with unique molecular contents and therapeutic potentials. Neural stem cell (NSC)–derived EVs have tremendous therapeutic potential for PD because they have neuroprotective and regenerative properties. Because they can cross the blood–brain barrier, NSC-EVs can deliver bioactive cargo like miRNAs, proteins, and lipids that modulate neuronal survival, mitigate oxidative stress, and suppress neuroinflammation [117]. Preclinical models demonstrate that NSC-EVs block intracellular ROS, downregulate apoptotic pathways, and protect dopaminergic neurons. They also inhibit pro-inflammatory cytokine release, holding back glial overactivation and inflammatory damage [118]. Interestingly, NSC-EVs contain specific miRNAs such as miR-182-5p, miR-183-5p, miR-9, and let-7 that promote neurogenesis, neuroprotection, and immune homeostasis. In 6-hydroxydopamine (6-OHDA) PD models [119], NSC-EV treatment inhibits dopaminergic neuron loss and improves motor performance. Compared to MSCs–secreted EVs, NSC-EVs have greater potential to promote neuronal differentiation, with less immunogenicity and lower tumorigenesis risk [120]. Their potential therapeutic application can also be enhanced further by the use of engineering approaches for the targeted delivery, with intranasal delivery being an effective mode to deliver brain targeting. Combined, NSC-EVs are a promising cell-free regenerative therapy for PD with potential to offer neuroprotection, pathological process control, and potential neuronal replacement support with fewer safety and ethical concerns than stem cell transplantation [121].

Stem cells from human exfoliated deciduous teeth (SHED)-derived EVs may have some advantages over EVs from MSC and NSC for PD treatment [122]. SHED-EVs are cranial neural crest cell-derived and possess higher proliferative potential and neuronal differentiation ability than MSC-EVs, hence enhancing their ability to target the repair of dopaminergic neurons [123]. They are supplemented with specific neurotrophic factors, growth factors, and anti-inflammatory molecules that may be more neuron-protective and regenerative and may be readily and ethically obtained from naturally shed teeth—a more accessible source than bone marrow or adipose-derived MSCs [124]. In contrast to NSC-EVs, SHED-EVs avoid ethical issues relating to embryonic or fetal origins and are simpler to harvest and expand for clinical application. Their cargo encompasses neural crest origin, having a broad reparative and anti-apoptotic action of specific value for dopaminergic neuron resilience in PD [125]. Considered collectively, the relative ease with which they are harvested, robust neurotrophic and immunomodulatory character, and lineage-specific advantages suggest SHED-EVs might be more advantageous or at minimum highly complementary to MSC and NSC EVs for forward PD therapy [126]. Intranasal delivery of SHED-EVs in preclinical PD studies has shown reduced loss of dopaminergic neurons within the substantia nigra, improved motor function, and lowered markers of oxidative stress [127].

Meanwhile, astrocyte- and microglia-released EVs can potentially serve as both disease mediators and therapeutic agents [128]. EVs from healthy astrocytes contain antioxidant enzymes (catalase, superoxide dismutase), glutamate transporters, and growth factors that can counteract oxidative stress and excitotoxicity [129]. Experimental therapeutic approaches employed glial EVs transduced with anti-α-synuclein antibodies or anti-inflammatory drugs to reduce aggregation and immune activation in PD models [130].

In addition, EVs derived from endothelial progenitor cells, induced pluripotent stem cells (iPSCs), and embryonic stem cells (ESCs) have been tested in various neurodegenerative models, including PD [131,132]. The vesicles may deliver enzymes like catalase, dopamine, or precursors to dopamine biosynthesis, as well as miRNAs that target autophagy, mitochondrial repair, and synaptic regulation pathways. Another strategy involves genetic modification of the parent cells to load EVs with selected therapeutic RNA or protein cargo for enhanced disease-targeting [133]. However, the heterogeneity of EV sources allows for advantages to be tailored to desired effects, such as neuroprotection, anti-inflammatory properties, or the removal of α-synuclein, potentially also with diminished immunogenicity or reduced moral concern [134]. However, there are challenges with scalable production, uniform characterization of cargo, controlled release, and avoidance of harmful pro-pathogenic signaling [135,136]. Comparative studies between EV types remain the exception, and thus whether the optimum balance of efficacy, safety, and clinic feasibility for PD is supplied by which source remains to be determined.

Meanwhile to their therapeutic potential, EVs hold tremendous potential as diagnostic biomarkers for PD [137]. EVs from plasma, serum, cerebrospinal fluid, saliva, and urine could carry disease-linked cargo including α-synuclein, DJ-1, LRRK2, mitochondrial DNA, and some microRNAs, which can be detected using techniques like immunocapture, next-generation sequencing, or Raman spectroscopy [138]. These biomolecules suggest active neurodegenerative mechanisms and are a minimally invasive way of the early diagnosis of PD, differential diagnosis, and monitoring of disease. While their direct therapeutic application is still in its early stages, evidence from stem cell EV therapies suggests that plasma and other EVs may hold future promise as dual-purpose diagnostic and therapeutic tools in PD [139].

Theranostic EVs are a bipotential strategy against PD that combines therapy and diagnosis on the same platform [140]. EVs derived from NSCs and MSCs can deliver rescue cargos such as miRNAs, proteins, and even mitochondria to decrease oxidative stress, inhibit α-synuclein fibrillation, and modulate neuroinflammation, offering a cell-free targeted therapy [141]. In parallel, EVs in urine, CSF, or blood release pathological miRNAs and proteins as minimally invasive biomarkers for early disease diagnosis, monitoring of disease, and discrimination between subtypes. By merging both diagnostic and therapeutic functionalities, theranostic EVs open up avenues for individualized treatment protocols in PD although still at the preclinical stage, these dual-functional EVs hold great promise for the future of precision medicine in PD meanwhile optimizing targetability, standardizing the procedure, and ensuring safety in the clinic remain challenging [142]. Table 2 summarizes the various non-MSC EV sources for PD therapy.

## 6. Clinical Trial of EVs for PD

EVs, especially those originating from neural or stem cell sources, have drawn an immense amount of attention in PD research due to their role in intercellular communication and cargo delivery. The majority of the ongoing clinical trials with EVs in PD are focused on appreciating their application as diagnostic and prognostic biomarkers rather than as therapies [143]. Various studies have proven that EVs, particularly α-synuclein-bearing EVs derived from neurons, can classify PD from other parkinsonism subtypes [144]. Moreover, concentrations of α-synuclein and other pathologic proteins in EVs isolated from CSF, plasma, and saliva have produced promising correlations with disease course and severity [144,145].

Numerous registered clinical trials observational in nature, seeking to determine the diagnostic value of EVs in biofluids, exist [146]. For example, the trial NCT05320250 investigates salivary EVs for PD biomarker identification [147], it was building on previous research in which Raman profiling of blood EVs correlated with clinical ratings (UPDRS III, Hoehn and Yahr), this approach aims to identify disease-specific biochemical fingerprints rather than employing a single protein biomarker like α-synuclein [148]. Previous research from the same team and others has shown moderate to high sensitivity and specificity of discrimination against neurodegenerative disease, though this trial hopes to provide formal confirmation [149]. In addition, NCT03775447 investigates whether CSF-derived EVs can mirror PD-related change [150]. These studies identify a growing interest in non-invasive and valid biomarkers that will help to diagnose early, monitor the disease, and evaluate treatment response [151]. No trial currently exists that has progressed to the application of EVs as a direct therapeutic intervention in PD patients. On the other hand, preclinical studies are solid evidence of the therapeutic potential of EVs, particularly those derived from MSC-EVs and NSC-EVs [152]. A recently published systematic review of 13 randomized preclinical studies reported noteworthy improvements in motor coordination, limb function, and neuronal survival using MSC-EV therapy [153]. Notably, the therapeutic effects were evident after two weeks of treatment and lasted for up to eight weeks. In addition to native EVs, bioengineered EVs bearing therapeutic payloads such as dopamine, catalase, or α-synuclein-targeting siRNA demonstrated enhanced activity in preclinical models of PD [154]. These findings affirm the application of EVs not just as neuroprotective treatment but also as targeted drug-delivery nanocarriers in neurodegenerative disorders.

## 7. Unresolved Mechanisms of EVs in PD

Despite increasing evidence for therapeutic and pathogenic roles of EVs in PD, key mechanistic issues remain unresolved. One such gaping hole is how EVs have neurodegenerative versus neuroprotective effects. While EVs can transfer pathological α-synuclein aggregates along with regulatory cargo such as cytokines and microRNAs, the molecular determinants of cargo selection, intracellular trafficking, and intercellular transfer are presently unknown [154]. Experimental evidence implicates EVs in α-synuclein transmission but remains to be tested and speculative for the sequence of successive events from EV uptake to intracellular aggregation, axonal dysfunction, mitochondrial injury, and neuron killing in vivo. Furthermore, it is unknown whether EV-induced neurotoxicity is due to α-synuclein aggregates alone or synergistically by other EV components [155].

Therapeutic EV design also makes things more complex. Symbiosis between endogenous EV cargo and exogenously supplied molecules such as siRNAs, proteins, or drugs could result in off-target effects or interfere with endogenous functions [156]. Targeted CNS delivery remains a significant challenge; although surface engineering strategies (peptide ligands, enzyme-mediated modifications, or genetic engineering) have been employed to enhance brain targeting, they generally compromise EV stability, reduce loading efficiency, or enhance risk for immunogenicity [157]. Low native EV yields, high cost of production, and lack of standard isolation and quality control protocols also hinder clinical scalability [158].

Mechanistic uncertainties also impede translation. EVs are credited with controlling α-synuclein aggregation, enhancing autophagy, reducing oxidative stress, and modulating immunity, but contradictory evidence on their uptake, biodistribution, and blood–brain barrier permeability limit reproducibility [159,160]. Preclinical outcomes are extremely heterogeneous across models, isolation methods, and dosing regimens. While MSC-derived EVs are generally neuroprotective, variable effects of NSC- and plasma-derived EVs occur, ranging from strong to no clearance of α-synuclein [161].

Clinical translation is in its infancy. The EV-based PD trials that exist are short-term, small-scale, and offer no data on long-term safety or efficacy. Issues related to immunogenicity, tumorigenicity, and off-target toxicities are still not addressed [162], particularly for repeated doses. The EV preparations used currently possess no specificity towards dopaminergic neurons, lowering potency while increasing risk of side effects. Absence of regulatory guidelines regarding quality control, potency assays, and release criteria also poses challenges for clinical progress [163].

The most important outstanding questions are which type of EV is optimal, under which dosing schedules and through which modes of delivery, and whether cargo within EVs will be stable in the circulation [164]. An important additional complicating factor is that no in vivo biomarkers exist to sensitively track EV function. Preclinical research utilizes most commonly acute toxin-based models that do not accurately represent the chronic, progressive nature of PD and, therefore, lack translatability [165].

Single-vesicle technologies, omics, and high-end imaging may be able to meet these challenges. Fluorescence, bioluminescence, super-resolution microscopy, and electron microscopy enable EV biodistribution and EV uptake to be followed, while omics technologies map EV cargo and identify stage-specific molecular features of PD [166,167]. Single-vesicle analysis, such as nanoscale flow cytometry and RNA/protein sequencing, can decipher EV heterogeneity, detect rare pathogenic subpopulations, and improve quality control for therapeutic production [168]. Together, these emerging tools offer a platform for improved mechanistic insight, biomarker discovery, and rational design of EV-based therapies for PD.

## 8. Emerging Nanocarrier Systems for PD Therapy

While EVs including MSC–derived EVs have attracted a lot of attention as natural delivery systems in PD therapy, an entirely new class of synthetic and bioinspired nanocarrier systems is emerging at a fast rate. These systems are designed to overcome the same basic therapeutic challenges as EVs such as BBB crossing, cell-specific targeting, and controlled delivery of bioactive cargo but offer greater flexibility in terms of composition, cargo nature, and engineering potential.

### 8.1. Lipid Nanocarriers

Lipid nanocarriers such as liposomes, solid lipid nanoparticles (SLNs), and nanostructured lipid carriers (NLCs) are gaining attention as having promise as platforms for therapeutic treatment of PD [169]. Their biological membrane structural resemblance, their high biocompatibility, and their tunable physicochemical properties make them particularly effective at crossing the BBB to the brain tissue and providing direct delivery of neuroprotective agents to the target tissue [170]. Besides facilitating increased bioavailability of drugs, these carriers also provide protection against enzymatic degradation in systemic circulation for therapeutic molecules that are sensitive to such, thereby improving the likelihood of achieving therapeutic levels at the target location [171]. Of these, SLNs have shown tremendous promise in preclinical PD models [172]. Composed of physiologically stable lipids solid at body temperature, SLNs are capable of entrapping lipophilic as well as hydrophilic drugs [173]. In rotenone-induced rat and zebrafish PD models, SLN preparations encapsulating neuroprotective compounds exhibited increased motor coordination, suppression of oxidative markers such as malondialdehyde, recovery of antioxidant enzyme activity, and suppression of α-synuclein aggregation with controlled release, stability, and biodegradability [174]. Similarly, S-carboxymethyl-L-cystine-loaded SLNs (SCSLNs) enhanced locomotion activity, grip force, and investigative behaviour in rat PD models, as well as reducing biomarkers of oxidative stress and preventing Lewy body formation [175]. Correspondingly, capsaicin extract-loaded SLNs demonstrated significant antioxidant activity in neuroblastoma cells, reducing reactive oxygen species induced by neurotoxic compounds [176]. These results point to the possibility of SLNs being more than drug carriers but also active neuroprotective compounds through synergistic interactions with their payloads. SLNs have also shown promise in increasing the delivery of conventional and investigational PD drugs. Glycol chitosan-enhanced dopamine-loaded SLNs had enhanced encapsulation efficiency and stability that made them suitable for intranasal delivery, a non-BBB-permeable pathway with rapid absorption in the brain [177]. SLNs and nanostructured lipid carriers, both oral and hydrogel, of ropinirole increased bioavailability and increased dopamine and glutathione levels while reducing lipid peroxidation in rat models [178]. This is a manifestation of the versatility of lipid-based carriers to diverse delivery routes and their aptitude for long-term, site-specific drug release. Natural bioactive compounds have also been efficiently incorporated into SLNs as PD therapy. Vitexin-loaded SLNs reduced oxidative stress, augmented antioxidant enzyme activity, and protected dopaminergic neurons from toxin-induced damage, while also preventing cognitive and mood dysfunction in mice [179]. Similarly, naringenin-loaded SLNs were found to have predictable release behaviour and neuroprotection against rotenone-induced PD in rodents, which suggests that they hold potential in slowing disease progression [180]. One of the most important factors determining the efficacy of lipid nanocarriers in PD is route of administration. Intranasal delivery of SLNs and NLCs has some advantages by bypassing the BBB by olfactory and trigeminal nerves, thereby avoiding first-pass metabolism and ensuring direct brain delivery [181]. Lipid nanocarrier encapsulation not only protects sensitive molecules from enzymatic degradation in the nasal cavity but also enhances mucoadhesion and prolonged release, resulting in greater brain accumulation and therapeutic effect [182,183]. Intranasal delivery of SLNs encapsulating neurotrophic factors such as GDNF has demonstrated enhanced behavioral recovery, reduced oxidative stress, and protection of dopaminergic neurons in models of PD in preclinical studies [184,185]. On the other hand, IV administration of SLNs or NLCs, while offering systemic availability, is restricted by the dense BBB, normally leading to compromised CNS bioavailability and increased or multiple dosing, except that the carriers are surface-engineered to enhance brain uptake [186]. Overall, comparative evidence indicates that intranasal delivery is better targeted to the brain, more bioavailable, and less systemically exposed than intravenous administration, establishing its promise as a non-invasive option for CNS drug delivery in PD [187].

Liposomes, by virtue of their new phospholipid bilayer architecture, can entrap both water-soluble and hydrophobic drugs; thus, they are highly versatile for the treatment of PD [188] due to their ability to encapsulate hydrophilic and hydrophobic drugs, their biocompatibility, and their ability to penetrate the brain through the BBB. This is significant in PD therapy, where conventional drug delivery does not work due to poor brain penetration and systemic side effects [189]. By manipulating liposomal composition, size, and surface modification, researchers have achieved successful enhancement of brain targeting, prolonged circulation time of the drug, and satisfactory sustained release of therapeutic agents, thereby maximizing clinical benefit at minimized toxicity [190]. One of the first demonstrations of liposome-mediated PD treatment was intrastriatal delivery of dopamine-loaded liposomes in the PD model rat [191]. This approach provided long-term dopamine release for over 40 days, which produced partially refilled levels of dopamine and improved motor behavior. Liposomal formulations have progressed significantly since then [192]. For instance, newer formulations based on amyloid precursor protein-derived peptides have been shown to have better brain penetration and higher striatal levels of dopamine, with implications that wise peptide conjugation can improve BBB transport and dopaminergic replacement [193]. Similarly, dopamine-delivery liposomes were PEGylated in order to target brain tissue with up to a sevenfold increase in delivery over normal liposomes, showing the potential of targeted surface modification [194]. Beyond direct replacement of dopamine, liposomes have been used to deliver neuroprotective and disease-altering agents. Astaxanthin-loaded lactoferrin-conjugated liposomes demonstrated potent antioxidant and anti-inflammatory effects, inhibiting oxidative stress, neuroinflammation, and dopaminergic neuronal loss in MPTP-induced models of PD [195]. In the same way, angiopep-2-functionalized liposomes loaded with echinacoside exhibited increased brain uptake and therapeutic activity to avoid oxidative stress, dopaminergic neuron loss, and motor dysfunction in mouse models of PD [196]. Other new systems, such as RVG29-functionalized liposomes containing a dopamine derivative (BPD), have enabled targeted neuronal delivery with improved therapeutic effect and without systemic toxicity. These cases open up the potential of liposomes as dual neuroprotective delivery vehicles and symptomatic relief carriers [197]. Multifunctional and diagnostic applications are increasingly on the rise for liposome technology. Magnetoliposomes, as examples, combine treatment delivery with magnetic responsiveness and potential for externally controlled navigation and even theranostic (therapy + diagnostic) uses [198]. Zwitterionic nanoliposomes have also been shown to be capable of interfering with the beta-sheet aggregation of α-synuclein, lowering neurotoxicity and reactive oxygen species without compromising neuronal calcium homeostasis [199]. Their disease-modifying activities position liposomes not only as a drug delivery vehicle but also as active agents in preventing PD progression.

NLCs are a highly advanced and versatile drug delivery system for the treatment of PD offering remedies to most of the drawbacks of conventional formulations. These lipid nanocarriers are a blend of solid and liquid lipids, which improves the drug loading, stability, and sustained release [200]. One of their major benefits is that they have the ability to trap hydrophilic and lipophilic drugs at the same time with improved entrapment efficiency and long-term stability. NLCs can be controlled for various administration routes like nasal, intravenous, oral, and even local dermal delivery, where both local and systemic treatments can be employed [201]. This flexibility enables NLCs to overcome significant limitations in PD therapy such as drug poor solubility in certain instances, fast metabolism, poor brain penetration, and the necessity for repetitive dosing. Different preclinical studies have affirmed the potentiality of NLCs to increase drug bioavailability, prolong circulation time, and maximize brain accumulation of anti-Parkinsonian medications [202]. For example, nasally delivered selegiline hydrochloride-loaded NLCs released the drug continuously for 22 h, restored motor activity in rotenone-induced PD rats, and provided effective nose-to-brain delivery without invasive techniques [203]. Similarly, did levodopa co-drugs in NLCs, which exhibited increased stability to enzymatic degradation, retaining drug integrity for prolonged periods and providing controlled delivery, which potentially translates to less erratic motor control and fewer side effects [204]. Intravenously administered NLCs of apomorphine demonstrated efficient targeting to localized regions of the brain, retarded drug release, and potentiated therapeutic action compared to conventional solutions [205]. These findings emphasize the versatility of NLCs in optimizing pharmacokinetics and therapeutic efficacy through designing delivery routes and formulation. NLCs, beyond conventional dopamine-replacement therapy, have also been explored for neuroprotection and disease-modifying strategies. NLCs modified with lactoferrin or peptides may be used to deliver neurotrophic factors like GDNF for increased survival of dopaminergic neurons as well as improved motor recovery [206]. Ropinirole-loaded polyethylene oxide and polypropylene oxide block copolymer-based NLC hydrogels have shown 24 h sustained release, acceptable stability under different storage conditions, and augmented neuroprotection with an augmented level of dopamine, reduced oxidative stress, and improved behavioral performance in PD models [207]. In addition, NLCs that encapsulate antioxidant drugs like alpha-mangostin have been found to suppress neuronal loss in multiple regions of the PD-infected brain, holding potential for the treatment of oxidative stress–induced neurodegeneration [208]. These observations suggest that NLCs can exert symptomatic as well as disease-modifying effects, making them of extreme value in multi-dimensional PD therapy. Of special promise is the use of NLCs for nose-to-brain delivery, which bypasses the BBB entirely through the olfactory and trigeminal nerve pathways. This route not only minimizes direct brain uptake but reduces systemic exposure to the lowest level possible, thereby reducing peripheral side effects. Intranasal gelatin-based NLCs have been used in research for delivery of basic fibroblast growth factor (bFGF) and shown efficient brain targeting, neuroprotection, and functional recovery in PD rat models without causing nasal tissue damage [209,210]. It is in line with the growing emphasis on non-invasive and patient-compliant drug delivery systems that improve compliance and quality of life. Secondly, NLCs are capable of being multifunctional and can be engineered for therapeutic payload and imaging agent delivery for the simultaneous treatment and imaging of diseases offering theranostic PD opportunities [211]. Table 3 shows the comparison of liposomes, SLNs, and NLCs as lipid-based nanoformulations for PD management.

### 8.2. Polymeric Nanoparticles

Polymeric nanoparticles (PNPs) have been of significant interest as a possible platform for the treatment of PD, largely due to their ability to address the principal problems of drug delivery to the brain [212]. Treatments for PD are typically hampered by poor CNS bioavailability of drugs and system side effects induced peripherally due to high drug concentrations. PNPs based on biocompatible and biodegradable polymers can encapsulate therapeutic drugs, shield them from degradation, and release them in a controlled fashion, enhancing therapeutic performance while minimizing side effects [212,213]. Their greatest advantage may be that they are able to cross or circumvent the BBB by surface engineering or targeting using a ligand, enabling direct targeting of dopaminergic neurons and other brain regions involved [214]. There are numerous preclinical studies demonstrating symptomatic and neuroprotective actions of PNPs in PD models [215]. Retinoic acid-loaded polymeric nanoparticles (RA-NPs), for instance, have been reported to maintain dopaminergic neurons, reduce neuronal degeneration in the substantia nigra, and enhance the transcription of transcription factors such as Pitx3 and Nurr1, both of which are crucial in the development and survival of dopaminergic neurons [216]. Other derivatives, such as ginkgolide B-loaded nanoparticles, exhibit extended release, improved brain penetration, improved locomotor activity, and improved levels of dopamine in PD animal models [217]. The results show the potential of PNPs to go beyond the control of symptoms and even change the course of the disease through neuroprotective mechanisms. PNPs are also versatile with drug selection and administration routes [218]. They are able to encapsulate low molecular weight substances like levodopa and dopamine (for symptom management) and sophisticated therapeutics like neurotrophic factors or gene therapy agents (for disease modification). Encapsulation of levodopa into PNPs has been shown to improve the stability of levodopa, enhance targeted delivery to the brain, and reduce peripheral side effects by reducing systemic exposure to dopamine [219]. In addition, polymeric carriers are also conceived for alternative routes of administration including intranasal, transdermal, and intravenous administration, ensuring flexibility to improve patient compliance and pharmacological effectiveness [220]. Emerging trends with PNPs focus on targeted as well as multifunctional delivery systems. Nanoparticles conjugated with ligands, for example, can be prepared to target specifically receptors on the BBB or on neuronal cells, which will improve drug delivery specificity [221]. Nanocarriers, including neurotensin-polyplex systems, demonstrate the promise of direct gene delivery to dopaminergic neurons with instructions for neurotrophic factors to induce long-term neurorestorative effects [222]. Poly (lactic-co-glycolic acid) (PLGA) nanoparticles can restore impaired lysosomal function by lowering lysosomal pH in cellular models of PD [223]. These nanoparticles can be delivered intranasally or through intracerebral injection, demonstrating widespread brain diffusion and cellular internalization [224]. PLGA-based L-DOPA nanoparticles show improved efficacy and sustained motor function recovery in rat PD models compared to standard drug forms [223,224]. Additionally, combining PLGA microcarriers with genetically engineered mesenchymal stem cells overexpressing neurotrophin-3 promotes dopaminergic neuron differentiation, offering a potential complementary therapeutic strategy for PD [225,226]. These studies highlight the promising role of PLGA nanoparticles in enhancing drug delivery and addressing lysosomal deficits in neurodegenerative diseases, particularly PD. Polycaprolactone (PCL) has also shown promise in the fabrication of drug delivery systems to treat PD [227]. Rasagiline mesylate-loaded PCL microspheres showed prolonged drug release and improved behavioral and biochemical outcomes in a rat model [226]. Similarly, a levodopa-encapsulated PCL nanocomposite enhanced motor symptoms and neurochemical changes in a mouse model [228]. PCL/gelatin scaffolds combined with Beta-Boswellic Acid synergistically improved the differentiation efficacy of stem cells into dopaminergic neurons, which can be applied for cell therapy [229]. Additionally, PCL microspheres encapsulated glial cell line-derived neurotrophic factor (GDNF) with 25-day controlled release and maintained bioactivity, which has potential use in treating PD [230]. These research studies highlight the flexibility of the PCL-based platforms for application in various aspects of PD therapy, from drug delivery to cell-based therapies. Recent studies have examined the therapeutic application of chitosan-based therapy against PD. Intravenous administration of chitosan in an MPTP-induced mouse model reversed dopamine neuron injury, modified gut microbiota, and improved intestinal and blood–brain barrier function by minimizing acetate levels and inflammation [231]. FTY720-loaded chitosan nanocarriers showed neuroprotective activity by inhibiting phosphorylated α-synuclein and PP2A-mediated epigenetic modulation in vitro and ex vivo [232]. Chitosan of low molecular weight demonstrated neuroprotection against rotenone poisoning in Drosophila, with improved locomotion and survival [233]. In addition, chitosan nanoparticles linked with nerve growth factor, acteoside, and plasmid DNA exhibited neuroprotective effects in cellular and mouse models of PD, suppressing the behavioral disorders and restored dopaminergic neuron loss [234]. These findings suggest the application of chitosan-based approaches as potential targets for PD treatment.

### 8.3. Metallic Nanoparticles

Metallic nanoparticles (MNPs) have emerged as a sophisticated platform with immense promise to revolutionize the diagnosis and treatment of PD. Their unique physicochemical properties, including controllable size, high surface-area-to-volume ratio, and modifiable surface chemistry, make them ideal candidates to cross the BBB [235]. AuNPs, in particular, have attracted interest for their multifunctionalism as drug delivery platforms, imaging agents, and therapeutic agents in their own right. Their intrinsic “nanozyme” activity allows them to mimic antioxidant enzymes catalase and superoxide dismutase, which can detoxify ROS and thus alleviate oxidative stress [236,237]. Furthermore, green synthesis approaches using plant extracts enable the synthesis of biocompatible AuNPs that combine the therapeutic efficacy of phytochemicals and nanoparticle-mediated delivery, which can synergize neuroprotection with minimized toxicity [238]. Apart from gold, several other metallic nanoparticles have shown neuroprotective activity in neurodegenerative diseases. Silver, selenium, zinc oxide, and iron oxide nanoparticles have demonstrated multifaceted mechanisms of action, including protein aggregation inhibition, ROS scavenging, neuroinflammatory pathway modulation, and mitochondrial function regulation [239,240]. Zinc oxide nanoparticles (ZnO-NPs), for example, were shown to engage in multiple neuroprotective mechanisms relevant to PD, from stabilizing dopaminergic neuronal function to reducing oxidative and inflammatory insults [241]. Similarly, Bacopa monnieri-mediated platinum nanoparticles have been shown to restore dopamine, recover locomotor activity, and enhance antioxidant defenses in preclinical models of PD [242]. Some new structures such as chiral molecule-mediated porous copper oxide (Cu_x_O) nanoparticle clusters exhibit multifunctional enzyme-mimicking activities that are targeted against oxidative stress and neuronal death in PD [243]. MNPs also have great potential to enhance biomarker detection and enable earlier diagnosis of PD. Complex nanostructures can significantly increase the sensitivity and specificity of pathologic protein detection, like α-synuclein, the main component of Lewy bodies, in biological fluids [244]. The potential for early detection could enable timely intervention before extensive dopaminergic neuron loss. In addition, MNP-based systems can be engineered for dual theranostic functions, delivering therapeutics while enabling real-time imaging of disease progression. Nanoparticles surface-functionalized with ligands can selectively bind to receptors on neuronal or glial cells, enabling targeted drug delivery with normal tissue sparing [245]. Targeted drug delivery not only enhances drug efficacy but also reduces systemic side effects.

### 8.4. Carbon-Based Nanoparticles

Carbon-based nanoparticles (CBNPs) like graphene, carbon nanotubes, and fullerenes are highly promising therapeutic and diagnostic agents for PD. These nanomaterials demonstrate unique neuroprotective properties and can be employed as effective vehicles for drug delivery with the potential to traverse the BBB, previously limiting therapeutic access to areas of the brain [246]. For instance, recent studies have demonstrated that nitrogen-doped graphene possesses the ability to suppress α-synuclein amyloid fibrillation, a distinctive pathological hallmark of PD, and thereby potentially retard disease progression. This disruption results from strong interactions between the graphene nanostructures and α-synuclein aggregates that reduce protein compactness and fibril formation [247]. In addition, the surface features of nanoparticles are principal regulators of brain penetration ability and biological target interaction, underpinning the importance of careful surface engineering to achieve maximal brain delivery efficiency. PEG-functionalized carbon nanotubes, when loaded with dopamine, have shown in vitro and in vivo neuroprotective activity, including alleviating oxidative stress and inflammation in PD mouse models, alongside enhancing dopamine-related neuronal markers [248]. Nanomedicine approaches using CBNPs have also the potential to enhance the bioavailability of drugs and minimize adverse effects compared to conventional therapeutics, and they have the promise to act as theranostic agents [249].

### 8.5. Nanogels and Dendrimers

**Figure 3:**
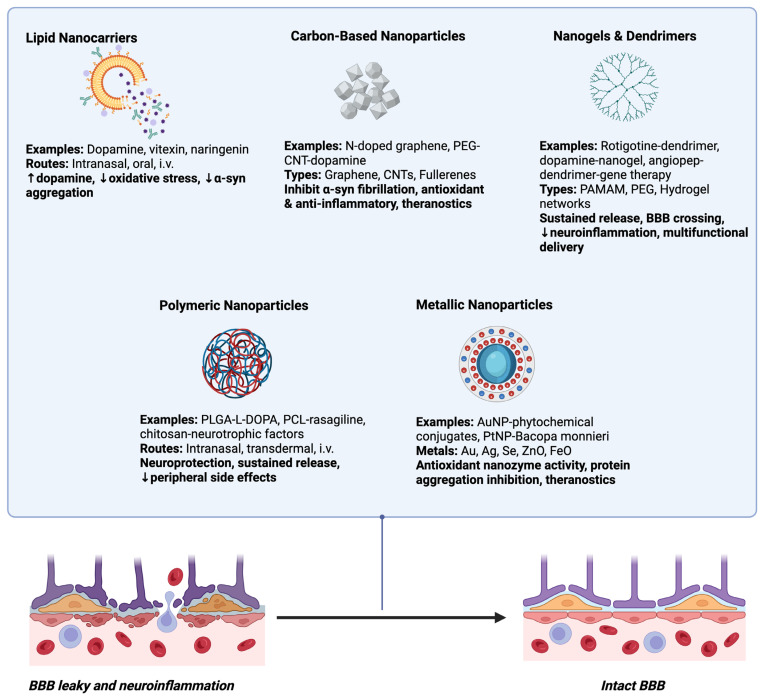
Classification of nanocarrier systems for PD therapy. (The arrow shows leading to).

Dendrimers and nanogels have emerged as novel and promising nanocarriers of therapy for PD, overcoming many shortcomings of conventional treatments. Dendrimers, by virtue of their highly branched, nanoscale structure, are able to cross the BBB and have shown the promise to interfere with the pathological aggregation of α-synuclein [250]. Studies by Ordonio et al. (2022) demonstrate that dendrimers not only ensure enhanced delivery of drugs like rotigotine and pramipexole to targeted brain regions but also increase bioavailability and extend drug release profiles, lowering dosing frequency and side effects, which is advantageous [251]. Moreover, dendrimers possess inherent anti-inflammatory and neuroprotective characteristics, as indicated by the researchers, such as the ability to modulate amyloid deposition and neuroinflammation, which are the major pathological processes involved in PD [252]. Similarly, nanogels, a cross-linked hydrophilic polymeric network that can encapsulate therapeutic molecules have shown outstanding potential for the delivery of dopamine and other drugs across the BBB [253]. For example, dopamine-loaded PVP/PAA nanogels exhibited effective BBB crossing and elicited significant enhancement of motor function along with the recovery of mitochondrial function in Parkinsonian rat models [254]. This points toward their possibility to exert disease-modifying instead of only symptomatic activity. The stimuli-responsiveness and biocompatibility of the nanogels also make them suitable for controlled and sustained release of drugs, which results in enhanced therapeutic efficacy without increased systemic toxicity. Recent advances include the preparation of fibronectin-complexed bioactive phosphorus dendrimers targeting specifically microglia to inhibit neuroinflammation and oxidative stress, leading to the normalization of dopamine levels and tyrosine hydroxylase expression in PD mouse models [255]. In another advancement, angiopep-conjugated dendrigraft poly-L-lysine nanoparticles have exhibited encouraging results for gene therapy, promoting neuronal recovery and motor activity in PD animal models [256]. These multifunctional dendrimer platforms have theranostic potential due to the convergence of targeted drug delivery and imaging diagnosis and contribute to personalized treatment approaches. Figure 3 shows clinically relevant nanocarriers for PD treatment.

Recent advances in the field of EVs and nanocarriers for treating PD are bringing about a revolution at a rapid rate by introducing new technologies to overcome traditional challenges such as inefficient BBB penetration, inefficient targeting, and unpredictable drug release [257]. Targeted or engineered EVs is one of the most promising directions, wherein surface engineering using ligands, peptides, or antibodies is being tailored to achieve cell-specific delivery of drugs and enhanced brain uptake [258]. Parallel to this, bioinspired functionalization of EV membranes or hybridization with synthetic materials improves their stability, half-life of circulation, and specificity of cargo loading [259]. At the nanocarrier side, novel surface engineering of lipid- and polymer-based platforms such as ligand conjugation, PEGylation, or peptide modification has significantly improved brain targeting, while multifunctional platforms now allow for simultaneous delivery of drugs, imaging, and even modulation of α-synuclein aggregation [260]. In addition, stimuli-responsive nanocarriers—pH-, enzyme-, or external trigger-sensitive like magnetic or light fields—enable spatiotemporally controlled delivery of neurorestorative or neuroprotective drugs with reduced systemic toxicity and improved therapeutic precision [261]. These novel approaches not only broaden EV and nanocarrier therapeutic opportunities but also provide theranostic opportunities, which suit the trend of PD treatment towards personalized and disease-modifying. Table 4 shows overview of different classes of synthetic nanocarriers under investigation for PD therapy.

## 9. EVs Versus Synthetic Nanocarriers in PD Treatment

EVs and synthetic nanocarriers are both explored as delivery systems for PD and both possess merits of their own. EVs are low immunogenic, biocompatible, endogenous vesicles with intrinsic permeability through the BBB by receptor-mediated transport. They deliver complex biological cargo, including miRNAs, proteins, and enzymes, modulating neuroinflammation, oxidative stress, and α-synuclein deposition [262]. MSC-EVs used in preclinical models show long-term motor recovery and dopaminergic neuron preservation, while NSC-EVs cause neuron regeneration with low immunogenicity [263]. Compared with them, synthetic nanocarriers are better at designing flexibility, controlled release, and drug-loading characteristics. They have been used as drug carriers for neuroprotective drugs, gene therapies, and α-synuclein modulators but typically require surface functionalization to reduce toxicity, immunogenicity, and inefficient targeting [264].

Route of delivery is significantly crucial to therapeutic efficacy. Intravenous injection, while widely used in preclinical models, results in excessive accumulation in clearance organs (spleen, liver, lungs) with reduced brain bioavailability [265]. Intranasal delivery, by contrast, bypasses the BBB via olfactory and trigeminal pathways, enabling augmented brain accumulation and reduced systemic toxicity [266]. EVs delivered intranasally, like those carrying catalase or siRNA, exhibit broad neuroprotection in PD models [267]. Synthetic nanocarriers are being engineered for intranasal delivery, but there remain mucosal penetration and retention problems [268]. CNS targeting is poor through oral, intraperitoneal, or subcutaneous administration.

Biodistribution and stability also characterize the two systems. EV tropism is dependent on cell origin, and EVs derived from neural or stem cells are more CNS-affinitive. They possess prolonged circulation and natural pathways such as receptor-mediated transcytosis to traverse the BBB [269]. Nanocarriers are also opsonized and cleared rapidly unless surface engineered (PEGylation, ligand conjugation) for improved stability and brain targeting [270]. EVs are clinically appealing due to their safety and low immunogenicity but translation is hindered by scalability, reproducibility, and purification challenges [271]. Nanocarriers benefit from validated manufacturing platforms and wider clinical use in drug delivery, but with limited CNS efficacy owing to biological barriers [272].

In general, EVs offer biological activity, inherent biocompatibility, and multitarget modulation of PD pathology, while synthetic nanocarriers provide design control, scalability, and reproducibility. Future approaches will likely integrate both strategies through hybrid EV–nanocarrier systems that take advantage of the natural targeting of EVs and the drug-loading capability and tunability of synthetic nanocarriers. Table 5 highlights their biological origin, BBB penetration, cargo, mechanisms of action, targeting potential, biodistribution, safety, scalability, theranostic applications, clinical status, unique strengths, and major challenges.

## 10. Conclusions

Studies on EVs have revolutionized our understanding of PD etiology and treatment. EVs are double-edged swords, supporting the prion-like spread of pathologic α-synuclein but also offering a natural drug delivery vehicle for therapeutic intervention. MSC-EVs have demonstrated amazing neuroprotection in preclinical models through multiple mechanisms including the delivery of neurotrophic factors, modulation of inflammatory responses, and promotion of neuronal survival. Parallel developments in synthetic nanocarrier systems have provided complementary solutions to the problem of drug delivery to the central nervous system. These developments represent significant milestones towards the realization of efficacious treatments for PD aimed at the disease processes per se rather than symptom modification. This would combine the intrinsic neuroprotection and immunomodulation of MSC-EVs with the advantageous qualities of nanocarriers like enhanced drug loading, prolonged release, and targeting to the brain through surface modifications. The combination of these modalities would potentially overcome current shortcomings of EV scalability and heterogeneity and enhance the therapeutic efficacy in PD.” Further research on MSC-EV–nanocarrier hybrids may therefore represent a powerful next-generation therapy for disease modification and improved clinical response. Meanwhile, several fields call for more intense research efforts. First, the development of hybrid delivery systems that combine the biological advantages of EVs with the engineering flexibility of synthetic nanocarriers could provide better therapeutic means. Second, scale-up EV manufacturing through bioreactor technologies will be essential for clinical translation. Third, precision targeting strategies with the use of selective ligands need optimization for maximum delivery to target brain regions. Fourth, combination therapies addressing multiple pathological aspects of PD simultaneously could be synergistic. Finally, rigorous clinical trials are necessitated to determine the safety and efficacy of these novel therapeutic modalities in human patients. The integration of EV-based diagnostics with therapy could permit tailoring of treatment regimens, and exploration of the gut–brain axis could uncover new therapeutic targets. By surmounting these hurdles, the next decade can see breakthrough advancement in PD treatment with huge patient gain.

## Figures and Tables

**Table 1:** Therapeutic mechanisms of MSC-EVs in PD.

Mechanism	Key Components	Biological Effects
BBB Penetration	Tetraspanins, Integrins, CXCR4/CCR2	Cross BBB to reach substantia nigra & striatum
Neurotrophic Support	BDNF, GDNF	Dopaminergic neuron survival, neurite outgrowth, synaptic homeostasis
Antioxidant Defense	Catalase, SOD	Scavenge ROS, reduce oxidative stress
Pro-survival Signaling	PI3K/Akt, MAPK/ERK activators	Enhance neuronal repair and regeneration
miRNA Regulation	miR-7, miR-153, miR-124	- Suppress α-synuclein expression - Enhance autophagy - Reduce apoptosis
Immunomodulation	TNF-α/IL-6/IL-1β inhibitors	Reduce microglial activation, limit neuroinflammation
α-Synuclein Clearance	Autophagy-inducing miRNAs (e.g., miR-26a)	Promote degradation of pathological α-synuclein aggregates
Mitochondrial Repair	Mitochondria-stabilizing factors	Improve energy metabolism, reduce ROS

**Table 2:** Non-MSC EV sources for PD therapy.

EV Source	Unique Cargo/Properties	Therapeutic Effects	Delivery Route	Challenges
Neural Stem Cells (NSCs)	BDNF, GDNF, neural miRNAs	- Axonal regeneration - Mitochondrial restoration	Intravenous, intranasal	Limited scalability
SHEDs	Anti-apoptotic miRNAs/proteins	- Reduce dopaminergic neuron loss - Lower oxidative stress	Intranasal [91,92]	Cargo variability
Astrocytes	Catalase, glutamate transporters	- Counteract excitotoxicity - Antioxidant effects	Local injection	Pro-inflammatory potential
Microglia	Engineered anti-α-synuclein antibodies	Target α-synuclein aggregates, reduce inflammation	Experimental [96]	Dual mediator/therapist role
iPSCs/ESCs	Dopamine precursors, synaptic miRNAs	- Dopamine synthesis - Synaptic repair	Not yet standardized	Ethical concerns (ESCs)
Endothelial Progenitors	Angiogenic factors	BBB repair, neurovascular support	Under investigation	Low CNS targeting efficiency

**Table 3:** Therapeutic effects caused by the nanoformulation upon PD.

Feature	Liposomes	Solid Lipid Nanoparticles (SLNs)	Nanostructured Lipid Carriers (NLCs)
Structure	Spherical vesicles with one or more phospholipid bilayers	Submicron particles with a solid hydrophobic lipid core	Solid lipid core mixed with liquid lipids creating a less ordered lipid matrix
Drug Encapsulation	Encapsulate hydrophilic drugs in aqueous core and lipophilic drugs in bilayer	Encapsulate both hydrophilic and lipophilic drugs in solid lipid matrix	Higher drug loading capacity than SLNs due to disrupted lipid matrix from liquid lipid
Stability	Moderate stability but prone to leakage and fusion	More physically stable than liposomes but can undergo drug expulsion due to lipid crystallization	Improved stability over SLNs; liquid lipids reduce crystallinity and drug expulsion
Drug Release Profile	Often faster release, sometimes burst effect	Controlled and sustained release, dependent on lipid matrix	More controlled and modifiable release than SLNs, better long-term stability
BBB Penetration	Good BBB transport, can be surface-engineered for enhanced targeting	Efficient BBB crossing with potential targeting ligand modification	Effective BBB penetration with enhanced targeting potential
Biocompatibility	Highly biocompatible, mimics cell membranes	Biocompatible, made from physiological lipids	Biocompatible, with improved flexibility in composition
Production Complexity	More complex and costly, often requires organic solvents	Easier and scalable production with less solvent use	Similar to SLNs but improved formulation parameters
Use in PD Models	Delivery of neurotrophic factors (e.g., GDNF), dopamine agonists; enhances neuronal uptake via fusion	Delivery of dopaminergic drugs (e.g., levodopa, ropinirole), antioxidants; sustained neuroprotection	Improved delivery of neuroprotective agents with higher drug loading and stability; investigational in PD models
Clinical Potential	Historically favored but limited by stability	Increasingly favored due to stability and controlled release	Emerging as superior alternatives to SLNs due to better encapsulation and stability

**Table 4:** Summary of key structural features of synthetic nanocarriers, therapeutic applications, advantages, and limitations.

Nanocarrier Type	Key Features	Therapeutic Applications in PD	Advantages	Limitations/Challenges
Lipid Nanocarriers (Liposomes, SLNs, NLCs)	Membrane-like lipid bilayers or lipid matrices; can load hydrophilic and lipophilic drugs	Dopamine replacement, neurotrophic factors (e.g., GDNF), antioxidants, neuroprotective compounds	High biocompatibility, good BBB penetration, sustained release, versatile routes (IN, IV, oral); NLCs allow higher drug loading and stability	Liposomes less stable (leakage, fusion); SLNs risk drug expulsion; scalability challenges
Polymeric Nanoparticles (PNPs)	Biodegradable polymers (e.g., PLGA, PCL, chitosan); tunable drug release	Levodopa, dopamine, retinoic acid, neurotrophic factors, gene delivery	Controlled release, surface modification for BBB targeting, multifunctional (drug/gene/protein delivery), flexible routes (IN, IV, transdermal)	Manufacturing complexity; variability in drug encapsulation; long-term safety data limited
Metallic Nanoparticles (MNPs)	Gold, silver, selenium, zinc oxide, iron oxide; high surface reactivity	Antioxidant effects, α-synuclein inhibition, biomarker detection, theranostics	Intrinsic catalytic (“nanozyme”) activity, dual therapeutic and diagnostic potential, surface functionalization for targeting	Risk of toxicity/oxidative imbalance; clearance and long-term safety concerns
Carbon-Based Nanoparticles (CBNPs)	Graphene, carbon nanotubes, fullerenes; unique electrical and mechanical properties	Dopamine delivery, inhibition of α-synuclein aggregation, antioxidant/anti-inflammatory effects	Strong BBB penetration, potential disease-modifying activity, high drug-loading capacity, theranostic potential	Biocompatibility and toxicity concerns; functionalization needed for safe use
Nanogels and Dendrimers	Cross-linked hydrophilic polymers (nanogels) or branched polymers (dendrimers)	Dopamine delivery, pramipexole, rotigotine, gene therapy, microglia-targeted anti-inflammatory agents	High drug encapsulation, stimuli-responsive release, BBB penetration, inherent neuroprotective and anti-inflammatory effects, theranostic potential	Complex synthesis, stability issues, need for more clinical validation

**Table 5:** The comparative features of EVs and synthetic nanocarriers in PD therapy.

Feature	Extracellular Vesicles (EVs)	Synthetic Nanocarriers (Lipids, Polymers, Metals, Carbon, Nanogels)
Biological Origin	Naturally secreted by cells (MSC, NSC, SHED, glia, iPSC, etc.); carry proteins, miRNAs, enzymes	Artificially engineered using lipids, polymers, metals, carbon, or dendrimers
Blood–Brain Barrier (BBB) Penetration	Intrinsic ability via receptor-mediated transcytosis (integrins, tetraspanins, CXCR4/CCR2); effective intranasally	Limited natural BBB penetration; often requires surface modifications or disruptive methods
Cargo	Endogenous bioactive molecules (BDNF, GDNF, catalase, SOD, miRNAs regulating α-synuclein, apoptosis, autophagy)	Drugs (levodopa, dopamine agonists), growth factors, antioxidants, siRNA, proteins; loaded synthetically
Mechanisms of Action	Multi-modal: neuroprotection, immunomodulation, α-synuclein clearance, mitochondrial repair, synaptic plasticity	Mainly symptomatic relief (dopamine delivery), antioxidant/anti-inflammatory action; limited intrinsic biology
Targeting	Some natural tropism (e.g., neural-derived EVs → CNS); engineering possible but may affect stability	Targeting mainly via chemical/biological modification (ligands, peptides, antibodies)
Biodistribution	Source-dependent tropism; accumulate in CNS more effectively via intranasal route	Often cleared rapidly by liver, spleen, kidney; peripheral accumulation common
Safety & Immunogenicity	Generally low immunogenicity, good biocompatibility; long-term safety still under study	Material-dependent; some risk of toxicity, oxidative stress, or inflammation
Scalability	Low natural yield; heterogeneity, reproducibility, and regulatory standardization are major barriers	More scalable manufacturing; established protocols for large-scale production
Theranostic Potential	Can serve as both biomarkers (α-syn, DJ-1, miRNAs) and therapies (theranostic EVs emerging)	Mainly therapeutic or diagnostic, rarely both; theranostics mostly in experimental stages
Clinical Status	Preclinical efficacy in PD models; early-stage safety trials ongoing; no completed PD clinical trials yet	More advanced in drug delivery field; some CNS nanocarrier trials exist, but BBB remains a bottleneck
Unique Strengths	Natural biology, endogenous cargo, multi-modal mechanisms, BBB penetration	Engineering flexibility, reproducibility, controlled cargo loading
Main Challenges	Scalability, heterogeneity, incomplete mechanistic understanding, regulatory gaps	BBB penetration, toxicity, long-term safety, limited intrinsic therapeutic functions

## Data Availability

There is no data to support the findings of this review.
